# Q fever, a rare cause of secondary hemophagocytic lymphohistiocytosis

**DOI:** 10.3205/id000085

**Published:** 2023-12-06

**Authors:** Juan Francisco Nieves Salceda, Pablo Lozano Cuesta, Sara Hermoso de Mendoza Aristegui, Jonathan Fernández-Suárez, Claudia Madrid Carbajal, Marta María García Clemente

**Affiliations:** 1 Division of Respiratory Medicine, Hospital Universitario Central de Asturias, Spain; 2 Microbiology Department, Hospital Universitario Central de Asturias, Spain

**Keywords:** pneumonia, coxiella, hepatitis, pulmonary medicine

## Abstract

Hemophagocytic lymphohistiocytosis (HLH) is a rare syndrome in which *Coxiella burnetii* is a very infrequent etiology. We present the case of a 62-year-old male with progressive pulmonary infiltrates, fever, hepatitis, and bicytopenia despite broad spectrum antibiotics. A thorough clinical evaluation led to a high suspicion of *Coxiella burnetii* infection, subsequently confirmed through a positive serum polymerase chain reaction (PCR) analysis. HLH diagnosis was established based on the fulfillment of 5/8 diagnostic criteria, obviating the need for a bone marrow biopsy. Targeted antibiotic treatment and dexamethasone led to full recovery within two weeks, eliminating the need for stronger immunosuppressive therapy.

## Introduction

Q fever is a worldwide zoonotic disease caused by *Coxiella burnetii*. Infection occurs by inhalation of bacteria from air contaminated by excreta of infected livestock [1]. The disease has an acute phase where nonspecific febrile illness, hepatitis, or pneumonia are most frequent, and a chronic phase, occurring in less than 5% of cases, leading to endocarditis. Hemophagocytic lymphohistiocytosis (HLH) is a rare disorder characterized by an aberrant immune response that leads to a sepsis-like syndrome that may rapidly progress to terminal multiple organ failure. HLH can be classified as primary, predominantly affecting pediatric population and associated with genetic factors, or secondary, occurring in the presence of underlying conditions such as infections, malignancies, or autoimmune diseases (Table 1 (Tab. 1)).

## Case description

We report the case of a 62-year-old male, smoker of 34 packs/year, with a history of rural living and horse ownership and absence of prior disease history. The patient presented with a week-long history of asthenia and high-grade fever up to 40ºC. Clinical examination revealed jaundice and bilateral crackles on lung auscultation. Laboratory testing (Table 2 (Tab. 2)) demonstrated bicytopenia, elevated acute phase reactants (APR), transaminases, hyperbilirubinemia, and hypertriglyceridemia. Chest radiography revealed bilateral nodular infiltrates, while abdominal ultrasound confirmed hepatomegaly without splenic abnormalities.

**Figure 1:**
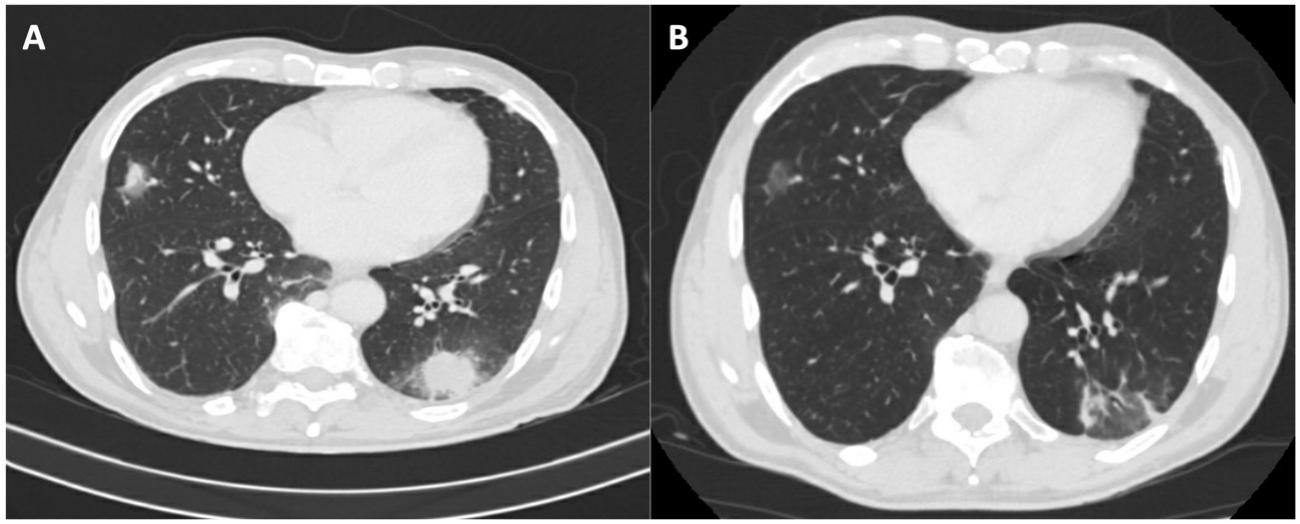
A: CT scan on patient admission with bilateral pulmonary infiltrates with halo sign; B: CT scan 3 months after discharge with reduction of pulmonary infiltrates

Upon admission, a chest-abdomen computed tomography (CT) scan demonstrated bilateral pulmonary infiltrates with a halo sign (Figure 1A (Fig. 1)). Blood and sputum cultures, as well as serological tests using indirect immunofluorescence (Vircel™) for *Mycoplasma pneumoniae *IgM, *Chlamydia pneumoniae *IgM, *Legionella pneumophila*, and *Coxiella burnetii *IgM/IgG phase II, returned negative results. Despite the initiation of ceftriaxone and levofloxacin therapy, the patient continued to experience fever and elevated APR, accompanied by deteriorating bicytopenia (hemoglobin (Hb) 9.4 g/dL, total leukocyte count (TLC) 1005/µL, neutrophils 900/µL, platelets 100,000/µL).

Considering the epidemiological context, symptoms, hepatomegaly, atypical pneumonia, and acute hepatitis, a high clinical suspicion of acute Q fever was raised. The diagnosis was confirmed through a positive serum real-time polymerase chain reaction (PCR) assay developed in-house (see Annex) and a later seroconversion of total IgM and IgG phase II antigens for *Coxiella burnetii* (Table 2 (Tab. 2)). Notably, the absence of preceding symptoms or positive IgG phase I antigens rendered chronic Q fever unlikely, and echocardiography ruled out endocarditis.

Peripheral blood flow cytometry (PBFC) was performed due to unexplained bicytopenia, revealing decreased natural killer (NK) cell activity. This finding, along with fever >38.5ºC, bicytopenia, hypertriglyceridemia, and elevated ferritin levels, led to the diagnosis of HLH, fulfilling 5/8 diagnostic criteria determined by the Histiocyte Society updated in 2004 [2]. Given the concomitant diagnosis of *Coxiella burnetii* infection, HLH was considered secondary to this pathogen [3]. 

The patient received a two-week course of doxycycline (100 mg/12h) and dexamethasone (60 mg/24h). Clinical improvement was observed, with resolution of fever within five days and a decline in APR levels (Table 2 (Tab. 2)). Following completion of treatment, the patient was discharged. 

At the three-month follow-up, the patient was asymptomatic, with negative PCR and positive IgG phase I/II serologies for *Coxiella burnetii*. A control CT scan displayed a reduction in pulmonary infiltrates (Figure 1B (Fig. 1)).

## Discussion

When encountering patients with atypical pneumonia and a history of contact with farm animals or products, including horses [4], the possibility of acute Q fever should be considered. Halo sign on CT scans can serve as a valuable diagnostic clue [5]. PCR testing for *Coxiella burnetii* shows higher sensitivity [6] in the early stage of an acute infection compared to serological tests, requiring a 7-to-15-day seroconversion time [1].

Doxycycline is the first-line treatment for an acute *Coxiella burnetii *infection, while fluoroquinolones represent an alternative antibiotic option. However, it is important to note that levofloxacin efficacy, which exhibits intracellular bacteriostatic effects against *Coxiella burnetii*, may be compromised in the presence of mutations in the quinolone-resistance-determining region [7], potentially explaining the lack of initial response to levofloxacin therapy. HLH is an abnormal hyperinflammatory state marked by excessive macrophage and T lymphocyte proliferation, potentially culminating in a fatal cytokine storm. Rising incidence rates, likely attributed to increased clinical awareness and improved diagnostic capabilities, are evidenced by England’s reported incidence of 4 cases per million in 2018, a quadruple rise from 2003 [8].

In adults, HLH secondary to infections, autoimmune disorders, and malignancies are predominant. An infrequent primary etiology described is adult onset HLH [9]. Hypomorphic mutations of known genes involved in familial HLH (PRF1, MUNC13-4, and STXBP2) play a role in the development of late-onset HLH [10]. In our case, the patient was not tested for HLH gene mutation.

The availability of a flow cytometry lab capable of performing PBFC obviated the need for invasive techniques such as bone marrow biopsy, mitigating potential complications in HLH diagnosis.

Treatment of adult HLH involves the use of immunomodulatory drugs including a combination of cyclosporin A, etoposide, or corticosteroids. Given the non-critical situation and directed antibiotic treatment, corticosteroids alone were used in our patient [11]. 

## Conclusion

HLH represents a life-threatening syndrome with heterogeneous clinical presentations, requiring prompt suspicion and intervention. Secondary HLH in adults often arises from underlying etiologies, demanding a meticulous search of underlying infections and malignancies. Among infectious pathogens *Coxiella burnetii* infection should be considered, despite being an infrequent cause of this syndrome.

## Annex

Primers are based in an in-house chromosome fragment (114 pb fragment: FQF: gctctcgccgaattcatca; FQR: gacgaagaagaatcgacagcct; FAM PROBE: gtttcagcttctttatcacg). The target of Coxiella Specific PCR is protein DNAa gene.

Results were confirmed with a subsequent PCR for 16S rRNA gene and sequencing according to Xu et al. [12].

## Notes

### Competing interests

The authors declare that they have no competing interests.

## Figures and Tables

**Table 1:**
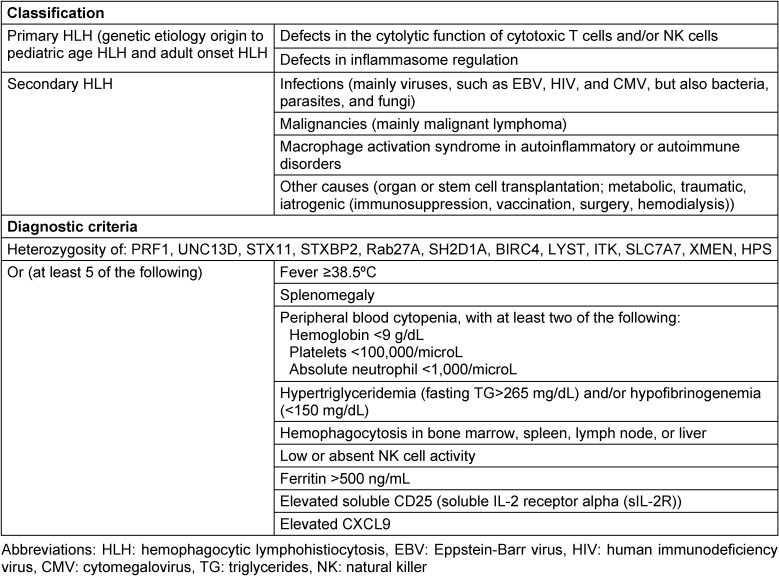
Classification and diagnostic criteria of hemophagocytic lymphohistiocytosis

**Table 2:**
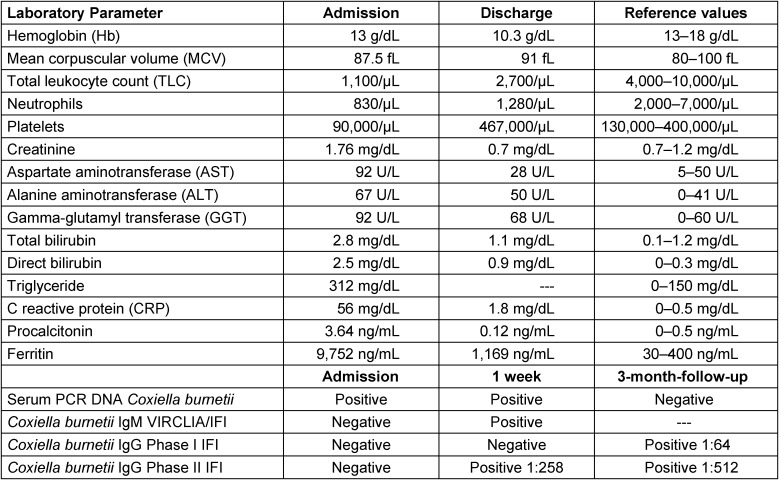
Serological and analytical values in patients’ admission and discharge
